# Evolving Perceptions of Feedback in Medical Communication Training

**DOI:** 10.1177/23821205251390750

**Published:** 2025-10-27

**Authors:** Hao Yu, Zhien Li, S Eleonore Köhler, Jeroen JG van Merriënboer, Maryam Asoodar

**Affiliations:** 1 Department of Educational Development and Research, Faculty of Health, Medicine and Life Sciences, 568602 School of Health Professions Education, 5211 Maastricht University, Maastricht, the Netherlands.; 2 Department of Anatomy and Embryology, Faculty of Health, Medicine and Life Sciences, 5211 Maastricht University, Maastricht, the Netherlands.

**Keywords:** simulated patient consultations, observer feedback, simulated patient feedback, video-annotated peer feedback, feedback discussion sessions

## Abstract

This exploratory study investigates how first-year international medical students’ perceptions of feedback may evolve during simulated patient consultations (SPCs). Over an 8-month period, 10 students participated in 4 SPCs and completed structured surveys after each session, ranking the value of 4 feedback sources: simulated patient feedback, observer feedback, video-annotated peer feedback, and group discussion sessions. Surveys combined Likert-scale ratings with open-ended questions to capture both quantitative and qualitative insights. The findings suggest a possible preliminary framework for feedback preferences. Early in the course, students most highly valued simulated patient feedback for its direct relevance to patient interaction. As the SPCs progressed, appreciation for observer feedback, video-annotated peer feedback, and group discussions increased. By the final SPC, group discussion sessions were frequently regarded as the most valuable, as they provided opportunities for collaborative reflection and shared learning. These preliminary observations point toward a framework in which learners’ feedback needs may shift from immediate, patient-centered input to more reflective and collaborative forms as they gain experience. The study highlights the potential importance of tailoring feedback strategies to students’ developmental stages. Given the small sample size and single-institution context, the results should be interpreted cautiously and considered hypothesis-generating. Future research with larger, multi-institutional samples is needed to test and refine this framework.

## Introduction

Effective communication is essential for quality healthcare, and its development is a critical component of medical education.^1,2^ Feedback plays a vital role in refining medical students’ communication skills by providing insights for improvement.^3^ According to Kluger and DeNisi's feedback intervention theory (FIT), feedback is most effective when it is specific, timely, and directly related to the task at hand.^4^ Hattie and Timperley's model further emphasizes that the impact of feedback depends on its focus, with task-level comments often most useful in the early stages of learning and more process-oriented feedback becoming valuable as learners gain experience.^5,6^

These theoretical perspectives suggest that feedback is not a one-size-fits-all tool, but that its effectiveness may depend on the learner's developmental stage.^3^ However, while much research has examined the immediate benefits of feedback, there has been less attention to how students’ preferences for different types of feedback may change over time. In particular, the literature has not fully addressed whether students’ evolving communication skills and growing experience influence the value they place on different feedback sources.

Simulated patient consultations (SPCs) offer a useful context in which to study this question. SPCs provide structured, realistic encounters in which students receive multiple forms of feedback—including input from simulated patients, observer comments, video-annotated peer feedback, and group discussion sessions.

Although each source may contribute differently at various stages of learning, little is known about how students’ appreciation of these sources develops across repeated consultations.

This study takes an exploratory approach to this gap. With a small sample of first-year international medical students, we investigate how students’ perceptions of feedback evolve across 4 SPCs conducted over an 8-month period. Rather than aiming to provide definitive conclusions, the study proposes a preliminary framework of feedback preferences, using the observed patterns as preliminary evidence. Our central question is: How does the perceived value of different feedback sources change as students’ progress through SPCs?

By focusing on the dynamic nature of feedback preferences, this study contributes to the literature in 2 ways: first, by providing preliminary evidence of how feedback needs may shift during early stages of medical training; and second, by offering a conceptual framework that can guide future research and the design of adaptive feedback strategies.

## Methods

### Feedback Design in SPCs

In our context, SPCs created realistic learning scenarios that considerably promoted the communication skills of medical students. Each SPC lasted between 30 and 40 min and covered topics such as SPC1: headache, SPC2: abdominal pain, SPC3: coughing, and SPC4: pain in the back, leg, or knee. Learners are prepared for these SPCs in a specialized lab equipped with consultation stations, examination beds, and hygiene facilities. Moreover, a video recording system was placed in the corner of the room to capture the SPCs, allowing students to upload the footage to an online learning portal and provide each other with video-annotated peer feedback.

Each SPC session included 3 participants: one student acting as the physician, one student serving as an observer, and one simulated patient. Simulated patients were well-trained and varied for each SPC interaction.

The participants were 10 students from an intact group enrolled in the international track of the medical program. They were invited and they voluntarily joined the study at the beginning of the course. After each SPC, the student doctor received feedback from the observer and the simulated patient. Approximately 2 weeks later, all 10 students would gather to discuss the feedback they had annotated on the video platform for each student doctor. This session was supervised by a tutor.

A survey study was conducted with 10 first-year medical students over an 8-month period, during which they participated in 4 SPCs. Surveys were administered after each SPC to assess students’ perceptions of 4 feedback sources: feedback from the simulated patients, the observer, peers’ video annotations, and feedback discussion sessions. The survey included both Likert-scale questions to rank the perceived value of each feedback source and open-ended questions to gather qualitative insights.

The survey instrument underwent a content validation process through expert review. Two independent experts in medical education and communication training reviewed the survey items for clarity, relevance, and potential sources of bias. Each expert first provided written feedback, which was then discussed with the research team in an iterative process. Based on their recommendations, 2 Likert-scale items were rephrased to improve readability, one open-ended question was modified to elicit more specific responses, and redundant wording was removed from the instructions. These revisions ensured that the survey achieved content validity and minimized the risk of ambiguous or leading questions. For example, one Likert-scale item asked participants to rate the perceived value of the following 4 feedback sources: (1) observer feedback, (2) feedback discussion sessions, (3) simulated patient feedback, and (4) video-annotated peer feedback. An example of an open-ended question was: “What aspects of the feedback from the simulated patient did you find most helpful?”

**Figure 1.**
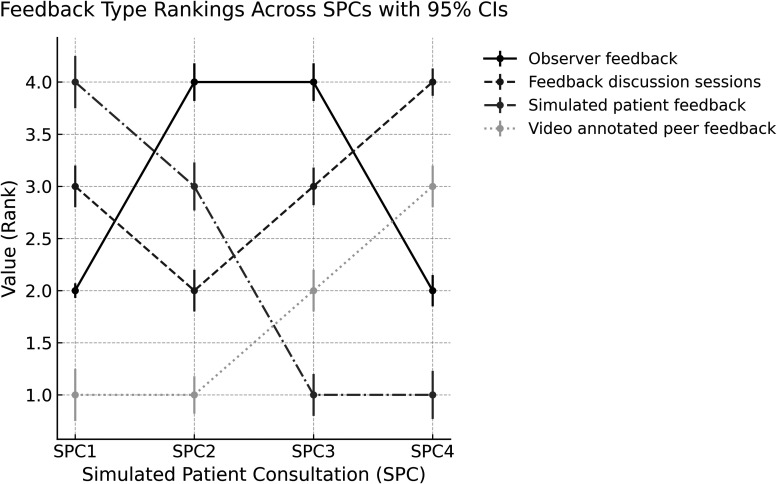
Students’ Perceived Value of Feedback Across 4 Simulated Patient Consultations (SPCs). Rankings (1 = Highest and 4 = Lowest) Represent the Relative Importance Students Assigned to Each Feedback Source at Each SPC.

Quantitative analysis: The Likert-scale responses from the 10 students, ranking the 4 types of feedback across the 4 SPC timepoints, were aggregated by summing the ranking values across all participants for each feedback source at each SPC. In addition, we applied nonparametric tests suitable for ordinal repeated measures, using the Friedman test to assess overall differences across SPCs and Wilcoxon signed-rank tests for pairwise comparisons. This approach produced a group-level hierarchy that reflected the total strength of preference, making higher cumulative scores represent greater perceived value. The aggregated data are presented in Figure 1.

Qualitative analysis: Open-ended responses were examined using thematic analysis. Two researchers independently coded the data and developed an initial codebook. The codebook was refined through iterative discussion until consensus was reached, and all coding decisions were documented to maintain transparency. Interrater reliability was established by calculating Cohen's κ, which yielded a value of 0.81, indicating strong agreement between coders. Representative student quotes were used to illustrate key themes, enhancing the credibility and rigor of the analysis.

## Result

This study examined how the students’ perceived value of feedback evolved over 4 SPCs. Figure 1 presents the ranking of 4 feedback sources, derived from survey results and open-ended responses provided by students following each SPC.

As shown in Figure 1, students’ preferences for feedback sources shifted over time. After the first SPC, simulated patient feedback was ranked highest (mean rank = 1.6, 95% CI [0.9, 2.3]), followed by feedback discussion sessions, observer feedback, and video-annotated peer feedback. Although this was the dominant group trend, some students consistently rated simulated patient feedback as most valuable throughout all SPCs, reflecting variability in individual learning needs. This pattern was confirmed by a Friedman test, which indicated significant differences in rankings across SPCs, χ²(3) = 15.2, *P* < .01.

Initially, simulated patient feedback was highly valued due to its direct relevance to patient interactions. For example, student 1 indicated that “My goal is to communicate effectively with patients. I believe that directly asking patients about my performance in SPCs is a very useful way to achieve this goal.”

In the second and third SPCs, observer feedback became the most valued source (mean rank = 1.8, 95% CI [1.0, 2.6]), followed by feedback by other feedback. Yet, a subset of students continued to prioritize patient feedback, showing that the transition was not uniform. Wilcoxon signed-rank tests confirmed the shift, showing a significant increase in observer feedback compared to SPC1 (*Z* = −2.45, *P* < .05). For example, student 5 indicating that “My observer knows me very well, because she is in the same pace with my learning, and she always supports me when I am nervous in performing or I make mistakes.”

Finally, in the fourth SPC, feedback discussion sessions were ranked as the most valuable feedback source (mean rank = 1.5, 95% CI [0.8, 2.2]), followed by video-annotated peer, observer, and simulated patients’ feedback (mean rank = 3.7, 95% CI [3.1, 4.3]). Wilcoxon signed-rank tests showed that group discussions significantly increased from SPC1 to SPC4 (*Z* = −2.67, *P* < .01), while simulated patient feedback decreased significantly (*Z* = −2.81, *P* < .01). For example, student 7 indicating that “Group discussions weren't always useful when we were all at the initial learning stage, as none of us really had much experience to share. We would just say things like, Oh, I'm nervous, or Don't worry, you did fine. However, as we've reached the final stage, we've come to rely heavily on the discussion sessions. Now, everyone can share their knowledge, experiences, mistakes, and solutions.” This quote captures how group-level changes in the rankings (Figure 1) corresponded with individual experiences of growing trust and shared expertise.

## Discussion

This exploratory study illustrates a potential preliminary framework of how medical students’ feedback preferences may change during SPCs. Rather than interpreting the observed shifts as definitive findings, we view them as exploratory evidence that illustrates how learners’ needs may evolve over time. In this framework, students initially valued direct, task-specific feedback—particularly from simulated patients—which aligns with FIT and its emphasis on actionable, task-related input in the early stages of skill development.^4^

As the students progressed, their focus appeared to shift toward feedback sources that encouraged reflection and collaborative learning, such as observer comments and group discussion.^7,8^ The increasing importance placed on group discussions suggests that learners may come to value opportunities for shared reflection and multiple perspectives as their confidence and competence develop.^5,6,9^ This interpretation resonates with feedback frameworks that highlight the progression from task-level feedback toward process- and self-regulation–oriented feedback.^4,10,11^

At the same time, several alternative explanations and potential confounding factors must be considered. Students’ growing familiarity with peers and increased psychological safety over time may have enhanced the perceived value of group discussions, independent of developmental progression. The tutor may also have become more skilled at facilitating discussions, and peers may have improved the quality of feedback they provided. These factors could have contributed to the observed patterns and made it difficult to disentangle developmental change from contextual influences.

### Theoretical Contributions

The primary contribution of this study lies in proposing a preliminary framework for feedback preferences. This framework suggests that learners may progress from relying heavily on direct, patient-centered feedback to appreciating more collaborative and reflective forms. By focusing on the relative importance of feedback sources over time, this model extends existing feedback theories and provides a foundation for generating hypotheses about how feedback strategies can be adapted to different stages of medical training.^10^

### Practical Implications

If feedback preferences evolve developmentally, educational strategies should adapt accordingly. In early training, simulated patient feedback may be particularly effective in helping students establish patient-centered communication. As learners advance, incorporating observer feedback and structured group discussion may support deeper reflection and skill refinement. Facilitated discussions, in particular, can provide a setting for integrating multiple viewpoints and applying feedback to refine communication skills. Technology-based platforms, such as video annotation tools, can further enhance reflective practice and promote professional growth.

### Limitations and Future Directions

The study's conclusions are constrained by several important limitations. First, the small sample size (*N* = 10) and single-institution context limit generalizability. Second, while social desirability bias may have influenced some student responses—given the possibility that participants shaped their answers to align with perceived expectations—we also observed that students were highly engaged in the learning process. They consistently treated the feedback mechanisms as opportunities for growth and made genuine efforts to provide constructive input to one another. These observations suggest that, although the potential for bias cannot be excluded, the data likely reflect authentic student perspectives. Third, several potential confounding variables cannot be ruled out. Students may have developed greater familiarity, trust, and psychological safety over time, making group discussions more valuable for social as well as developmental reasons. The tutor may also have become more effective at facilitating discussions, and peers may have improved in the quality of feedback provided. Because of the single-group design, it is not possible to separate the influence of developmental progression from these contextual factors. Fourth, although our results provide preliminary insights, their generalizability is restricted by the small, homogeneous sample and the single curricular and cultural context in which the study was conducted. Students’ feedback preferences may differ across cultural, linguistic, and institutional settings. Taken together, these limitations highlight that the current findings should be interpreted as exploratory and hypothesis-generating. Future research with larger, multiinstitutional samples and longitudinal designs is needed to test and refine the preliminary framework proposed here.

## Conclusion

This exploratory study provides preliminary insights into how medical students’ feedback preferences may evolve during SPCs. The data illustrate a possible preliminary developmental trajectory, in which students initially value direct feedback from simulated patients but gradually shift toward observer input and group discussion as their experience grows. The main contribution of this work is conceptual: it proposes a preliminary framework that highlights the potential for feedback preferences to change across training. These observations set an agenda for future research to validate and extend this framework across larger and more diverse student populations.
